# Municipally Educated Dentists

**Published:** 1918-11

**Authors:** 


### MUNICIPALLY EDUCATED DENTISTS

An interesting scheme, initiated by Mr. Harry Grove, of Walsall, for enabling secondary school students to become qualified dentists after a six-year period of training, has been taken up by the Education Committee of the Walsall Town Council. The scheme has been briefly described by Mr. W. Thompson Madin in the *British Dental Journal,* August 15th, as follows:—

"The Education Committee takes a certain number of picked students of the secondary schools, at the age of sixteen, and gives them as scholarships a complete dental training, paying all university and hospital fees, and receiving in return their half-time work, under the supervision of the school dentist in the School Dental Clinic. The Birmingham University has been able to arrange a time-table extending over six years, instead of the usual four, whereby the student can spend during one year the mornings in Birmingham and the afternoons in Walsall, and the next year *vice versa.* It will be observed that this scheme would accomplish four things of the utmost importance:—

"(1) It would provide for the treatment of children, by supplying school dentists with trained assistants.

"(2) It would produce an immediate increase in the number of dental students.

"(3) It would go far to provide the State with a steadily increasing flow of qualified dentists—a desideratum of the first magnitude.

"(4) It would obviate the double danger, which has been pointed out, of opening another door to irregular practice, and of creating the impression that operative dentistry can be taken up by those who have undergone but a fraction of the training demanded by the dental curriculum."—*Dental Record.*

